# Clinical and histopathologic features of canine tegumentary leishmaniasis and the molecular characterization of *Leishmania braziliensis* in dogs

**DOI:** 10.1371/journal.pntd.0007532

**Published:** 2019-07-16

**Authors:** Jamile Lago, Juliana A. Silva, Lairton Borja, Deborah B. M. Fraga, Albert Schriefer, Sergio Arruda, Ednaldo Lago, Edgar M. Carvalho, Olívia Bacellar

**Affiliations:** 1 Serviço de Imunologia, Universidade Federal da Bahia, Salvador, Bahia, Brasil; 2 Instituto Gonçalo Moniz–Fiocruz-Bahia, Salvador, Bahia, Brasil; 3 Instituto Nacional de Ciência e Tecnologia em Doenças Tropicais (INCT-DT), Bahia, Brasil; 4 Universidade Estadual da Bahia–Bahia, Brasil; Universidade Federal de Minas Gerais, BRAZIL

## Abstract

**Background:**

Cutaneous leishmaniasis (CL), caused by *Leishmania braziliensis*, is the most important presentation of tegumentary leishmaniasis (TL) in Latin American. While the role of dogs as reservoirs of *Leishmania infantum*, and the clinic features of canine visceral leishmanisis are well described, little is known about the importance of dogs in the transmission of *L*. *braziliensis* to humans. In the present study, we determine the frequency of *L*. *braziliensis* infection in dogs with cutaneous and mucosal ulcers in an endemic area of CL. We also describe the clinical manifestations and histopathologic features, and determine if the parasites isolated from dogs are genetically similar to those found in humans.

**Methodology:**

This is a cross sectional study in which 61 dogs living in an endemic area of CL and presenting ulcerated lesions were evaluated. Detection of *L*. *braziliensis* DNA by polymerase chain reaction (PCR) in skin biopsies, serology and leishmania skin test (LST) with soluble *L*. *braziliensis* antigen were performed. The clinical and histopathologic features were described, and we compared the genotypic characteristics of isolates obtained from dogs and humans.

**Principal findings:**

The sensitivity of the three tests together to detect exposure was 89% and the concordance between the tests was high. The skin lesions were most frequent in the ears, followed by scrotal sac. The PCR was positive in 41 (67%) of animals, and the lesions in the snout, followed by the scrotal sac and ears were the sites where parasite DNA was most detected. There were genotype **s**imilarities between *L*.*braziliensis* isolates from dogs and humans.

**Conclusions:**

The high frequency of *L*. *braziliensis* infection in dogs with ulcers and the similarities between the isolates of *L*. *braziliensis* and cutaneous leishmaniasis in dogs and humans in an endemic area of TL, raise the possibility of an important role of dogs in the transmission chain of *L*. *braziliensis*.

## Author summary

Cutaneous Leishmaniasis (CL) is the major clinical forms of tegumentary leishmaniasis. More than 1 million of people develop cutaneous leishmaniasis worldwide and *Leishmania braziliensis* is the main causal agent of the disease in Latin American. Wild and domestic animals play an important role in the maintenance of the parasites that are transmitted to humans by infected sandflies. There is no vaccine for leishmaniasis and control measures are not effective in part because it is not known the reservoirs of *L*. *braziliensis*. Here, we show that dogs, living in houses in an area of *L*. *braziliensis* transmission presented cutaneous and mucosal ulcers similar to the one presented by humans. In all the houses with canine CL there were active or past history of cases of human CL, and *L*. *braziliensis* isolated from dogs were genetically similar to the one from humans. These data are in favor of the participation of dogs in the transmission of *L*. *braziliensis* and may have a great importance in the control of the expansion of *L*. *braziliensis* infection.

## Introduction

Leishmaniasis is an antropozoonosis caused by the protozoa of the genus *Leishmania*, which is transmitted to humans by infected sandflies. Leishmaniasis is classified as visceral leishmaniasis (VL) and tegumentary leishmaniasis (TL). Approximately 20 *Leishmania* species may cause human disease. In the New World, VL is caused by *Leishmania infantum* and TL is mainly caused by *Leishmania Viannia braziliensis*, *Leishmania Viannia guaynensis* and *Leishmania mexicana mexicana* [1]. Foxes and the dogs are the most common wild and urban reservoir for *L*. *infantum*, and play a key role in the persistence of the disease [2]. Unlike VL, very little is known about the wild and urban reservoirs of parasites that cause TL in the New World. *L*. *braziliensis* is the most important causal agent of leishmaniasis in Latin America. Cutaneous leishmaniasis (CL) is the most frequent form of TL, occurring in more than 90% of patients. Additionally, about 3% of patients infected with *L*. *braziliensis* develop mucosal leishmaniasis and 2% disseminated leishmaniasis [3–5]. Amastigote forms of *Leishmania* have been documented in tissue from dogs, donkeys, horses and rats in endemic areas of *L*. *braziliensis*, raising the possibility of the role played by these animals in *L*. *braziliensis* transmission [6, 7]. In endemic areas of CL the exposure of dog to *Leishmania sp* have been documented by serologic tests, identification of amastigotes in biopsies of cutaneous lesions, or isolation of parasite in aspirated material from skin cutaneous ulcers [8]. However, the identification of the species of *Leishmania* was performed in only a few reports and there is a lack of studies describing the clinical and histopathologic features of the *L*. *braziliensis* infection in dogs. Unlike canine VL, a disease that is well characterized by different clinical forms, including asymptomatic, oligosymptomatic, and symptomatic clinic canine VL [9], there is no clinical and histopathologic characterization of TL in dogs.

Currently, the most studies included just a small number of animals evaluated and without identification of the parasite species [6,10]. Recently in the southern region of the state of Bahia, Brazil, a known endemic area for *L*. *braziliensis*, 560 dogs were analyzed for the presence of skin lesions of CL. Anti-*Leishmania* antibodies and a polymerase chain reaction (PCR) for detection of *L*.*braziliensis* DNA were performed. Specific primers were used to differentiate between *L*. *braziliensis* [11] and *L*. *infantum* [12]. In this study, 32 (6%) of the 560 dogs presented lesions suggestive of CL, and the PCR for *L*.*braziliensis* was positive in 54.7% of 413 skin biopsies obtained from dogs [13].

Genetic differences from isolates of *Leishmania* belonging to the same species have been widely documented [14–16]. Moreover, *L*. *braziliensis* is polymorphic and genotypic differences are associated with different clinical forms of the disease [17,18]. Thus, it is important in epidemiologic studies to determine if the species found in humans are similar to those found in animals, which may have the potential to participate in the transmission chain. Additionally, polymorphisms of *L*. *braziliensis* isolates from dogs have been detected [19]. However, no studies have been performed to evaluate if isolates of *L*. *braziliensis* from dogs are similar to the parasites isolated from humans. These data clearly indicate the need for studies that characterize canine cutaneous leishmaniasis and the role of dogs as a reservoir of *L*. *braziliensis*.

The aim of the present study was to determine the frequency of *L*. *braziliensis* infection in dogs with cutaneous and mucosal ulcers in an endemic area of human cutaneous leishmaniasis. Further, the study aimed to describe the clinical manifestations and the histopathology features of canine CL caused by *L*. *braziliensis*, and to determine if the parasites isolated from dogs are genetically similar to those found in humans.

## Materials and methods

### Ethical statement

This study was performed in strict accordance with the recommendations of Brazilian Federal Law on Animal Experimentation. The protocol was approved by the Ethics Committee for the Use of Animals in Research (CEUA license number: 015/2017) from the Gonçalo Moniz institute (IGM-FIOCRUZ). The owners of the dogs gave written informed consent prior to sample collection and the skin sampling procedures were performed under sedation. No adverse events were recorded during the sample collection.

### Study area

The study was conducted in the village of Corte de Pedra, Bahia, Brazil, where TL due to *L*. *braziliensis* is endemic. The village is characterized by isolated sites of secondary forest, with agricultural activities providing the main source of income for the majority of the population. Agricultural work increases exposure to *L*. *braziliensis* through increased contact with *Lutzomyia (Nyssomyia) whitmani* and *Lutzomyia (Nyssomyia) intermedia* sandflies, which are the vectors of *L*. *braziliensis* in the region [20].

### Study design

This is a cross sectional study to determine the frequency of canine leishmaniasis caused by *L*.*braziliensis* infection in dogs with ulcerated skin or mucosal lesions, to describe the clinical and histopathologic features of canine leishmaniasis caused by this species, and to determine the positivity of different diagnostic tests for *L*. *brazilensis* in these animals. Moreover, we compared genotypically the isolates of *L*. *braziliensis* from dogs with the isolates from human CL.

### Clinical examination and material collection

The 50 residences closest to the medical clinic of Corte de Pedra who had dogs with ulcerated lesions participated in the study. The selection of the dogs was based on the information by mouth about the presence of dogs with ulcerated lesions, initially in the houses close to the Health Post and later in four neighborhoods close to the Heath Post.

There were 61 dogs with lesions in the 50 houses. Initially, we explained to the dogs’ owners the objectives of the study and the informed consent form was read and signed by them. A questionnaire was answered about demographic characteristics of the animals, duration of the lesion and occurrence of previous or present cases of human disease in the house. The dogs were then immobilized, physical examination was performed and blood (8 mL) from the lateral saphenous vein was obtained.

After the area of the lesion was cleaned with alcohol and lidocaine for anesthesia was applied, a skin biopsy of the ulcer was obtained with a punch of 4 mm and added to Eppendorf tubes containing RNA Later Solution (Ambion, Life Technologies, Thermo Fisher Scientific, USA). In animals that had more than one lesion we choose the greatest ulcer to obtain the skin biopsy.

In about half of the animals, the biopsy was divided in two pieces being one added to formaldehyde and other to the RNA Later Solution.

### Diagnosis

#### Leishmania skin test (LST)

The *Leishmania* skin test was performed with soluble *Leishmania* antigen from the *Leishmania amazonensis* strain (MHOM / BR/2003/LTCP11245) and was obtained as previously described [21]. Each animal was injected intradermally with 100 μL volumes of the antigen solution on the posterior area of the abdomen. Forty-eight hours after the injection, the size of induration was measured. Skin reactions with induration size equal to or larger than 5 mm were considered positive.

#### Serological test to detect anti-*Leishmania* antibodies

An in-house ELISA, with *L*. *braziliensis* SLA, was performed as previously described [22–23].

#### Extraction of genomic DNA of *L*.*braziliensis* and determination of the species of *Leishmania* by Real-time PCR

In order to detect *L*. *braziliensis* DNA in the skin and muzzle of dogs, biopsy fragments from the border of the lesions were stored in the RNA Later Solution immediately after collection in field, and maintained at room temperature for approximately six hours until storage at 4°C in the laboratory. Two to three days later, the DNA was extracted from the skin and mucosal fragments using the DNA Purification Kit (Promega Co., USA), according to the manufacturer's recommendations. All dogs had their diagnosis later confirmed by detection of *L*. *braziliensis* DNA in PCR lesion biopsy specimens [24].

In all animals, PCR, ELISA for antibodies against *L*. *braziliensis* antigen and the skin test were performed.

#### Histopathology

To compare the histopathology features found in lesions from dogs with those already established in lesions from patients with CL [25, 26] we performed this analysis in the last 35 animals evaluated.

A cylindrical skin fragment obtained by 4 mm punch used for the skin biopsy was placed in a tube with formaldehyde and subsequently processed histologically in the histopathology service at IGM-FIOCRUZ Bahia, Brazil. The fragments were dehydrated and embedded in paraffin and stained by Hematoxylin and Eosina (HE). The microscopic analysis was performed in about three to four sections of each biopsy under the Nikon E 400 microscope with 10, 20 and 100X objectives. In the epidermis we evaluated the occurrence of hyperkeratosis, acanthosis and the presence or not of crust. In the dermis, the chronic inflammation mainly represented by lymphocytes, macrophages and plasma cells was classified as light, moderate or intense, based on the extension of the area of inflammation in the dermis by less than 30% (mild), ≥ 30 ≤50% (moderate), and greater than 50% (strong). The presence or absence of granulomas in the tissue and tissue necrosis were also evaluated. The detection of amastigotes was investigated under immersion with oil at 100X magnification. Analysis of at least 10 fields per sheets was performed. The images were obtained by scanning the 20X on the Olympus BX61VS microscope.

### Genotyping *L*. *(V*.*) braziliensis* from dogs and CL patients

Parasites were genotyped according to the haplotypes of polymorphic nucleotides in the locus CHR28/425451, previously shown to distinguish *L*. *(V*.*) braziliensis* strains in Corte de Pedra [27]. Primers 5´:TAAGGTGAACAAGAAGAATC and 5´:CTGCTCGCTTGCTTTC were used to amplify a 622 nucleotide-long segment in CHR28/425451 from parasite genomic DNA as previously described [27]. Amplicons were cloned using the Original TA Cloning Kit pCR 2.1 VECTOR (Invitrogen, Thermo Fisher Scientific Co., MA, USA), according to manufacturer’s instructions. Briefly, the amplicons were inserted by overnight ligation into PCR 2.1 plasmids, which were used for chemical transformation of competent DH5α *Escherichia coli*. Plasmid minipreps were generated from four recombinant bacteria colonies per study isolate [28]. Amplicon cloning was confirmed by digestion analysis, using Eco RI restriction endonuclease (Invitrogen). Plasmid inserts were sequenced by the Sanger method with primers complementary to the M13 vector sequences. Sequencing was performed at Macrogen Inc. (Seoul, South Korea). Mega 5.0 software [29] was used to align the sequences with the CHR28/425451 clones obtained from the panel of *L*. *(V*.*) braziliensis* parasites, in order to determine the SNP/indel haplotypes detectable in each study isolate.

### Sequence analysis

The study consisted of a panel of two groups of samples, 29 *L*. *braziliensis* isolates obtained from the dogs included in this study and 113 *L*. *braziliensis* isolates obtained from human beings in 2008–2011 periods.

In order to check whether *L*. *braziliensis* strains circulating among dogs in Corte de Pedra might be shared by human CL patients in that region, the 600 base pairs long locus that starts at nucleotide position 425,451 on the parasite’s chromosome 28 (i.e. CHR28/425451) was PCR amplified, cloned, sequenced and compared across a panel of isolates of dog or human origin. For analysis of the nucleic acid sequences of the polymorphic locus CHR28/425451, the consensus sequence at the locus explored from the CL sample (i.e., 2008–2011) was first determined, which was used to compare the different *L*. *braziliensis* samples obtained from 29 animals. Then, the sequences were analyzed for the identification of the occurrence of *SNPs* and/or *indels* for the identification of polymorphism alleles. We defined polymorphism as a single difference between the sequences of the evaluated isolates and polymorphic allele as a linear DNA sequence of the locus studied, detected in more than one clone per parasite isolate, and in more than one isolate of *L*. *braziliensis* in our study sample. The frequency of distinct alleles was determined in each isolate of *L*. *braziliensis* obtained from the dog biopsy for the locus CHR28 / 425451. The alignment between the isolates from dogs and humans showing the polymorphisms is presented in S1 Fig.

### Statistical analysis

The distribution of variables such as age, duration of disease (years), number of lesions and site of injury were expressed by mean and standard deviation and were analyzed by the student's T-test. The Chi-square and Fisher's exact tests were used for comparisons of proportions between groups. The comparative analyzes of the diagnostic tests were performed by the Kappa Index of concordance. A p value of <0.05 was considered statistically significant.

## Results

The 61 dogs with skin lesions enrolled in this study lives in 50 houses. Twenty animals (33%) presented more than one cutaneous lesion and 6 (9.8%) had also mucosal leishmaniasis. The demographic and clinical features according to the positivity of the PCR are presented in (Table 1). The percentage of males was higher in the group with positive PCR (p<0.008) but there was no difference between age, duration of illness and number of lesions with regard to the positivity of the PCR. The site of the largest lesion in those animals with a positive PCR was in the scrotal sac, followed by the ears and in six animals the lesions were in the muzzle. The PCR was positive in the lesion tissues of 41 (67%) of the 61 animals.

**Table 1:** Clinical and demographic features of 61 dogs with ulcerated skin or mucosal lesions in the village of Corte de Pedra.

Demographic and Clinical Features			
	PCR positive	PCR negative	P value
	N = 41	N = 20	
**Age (years), mean, (SD)**	5.7 ± 3.0	5.0 ± 3.6	0.4
**Age (years)**			
<5	16 (45%)	11 (55%)	ns
>5	25 (55%)	9 (45%)	
**Sex**			
Male	36 (88%)	11 (55%)	
Female	5 (12%)	9 (45%)	0.008
**Duration of lesion**			
<6 months	10 (24%)	9 (45%)	
6–2 years	22 (54%)	9 (45%)	ns
>2 years	9 (22%)	2 (10%)	
**Number of lesions**			
1	28 (68%)	14 (70%)	
1–3	8 (20%)	5 (25%)	ns
>3	5 (12%)	1 (5%)	
Site of the ulcer^*^			
Scrotal sac	17 (41%)	4 (20%)	
Ears	12 (29%)	12 (60%)	0.04
Muzzle	6 (15%)	0 (0)	
Others	6 (15%)	4 (20%)	

SD = Standard deviation

* Sites of the largest ulcer

**Fig 1:**
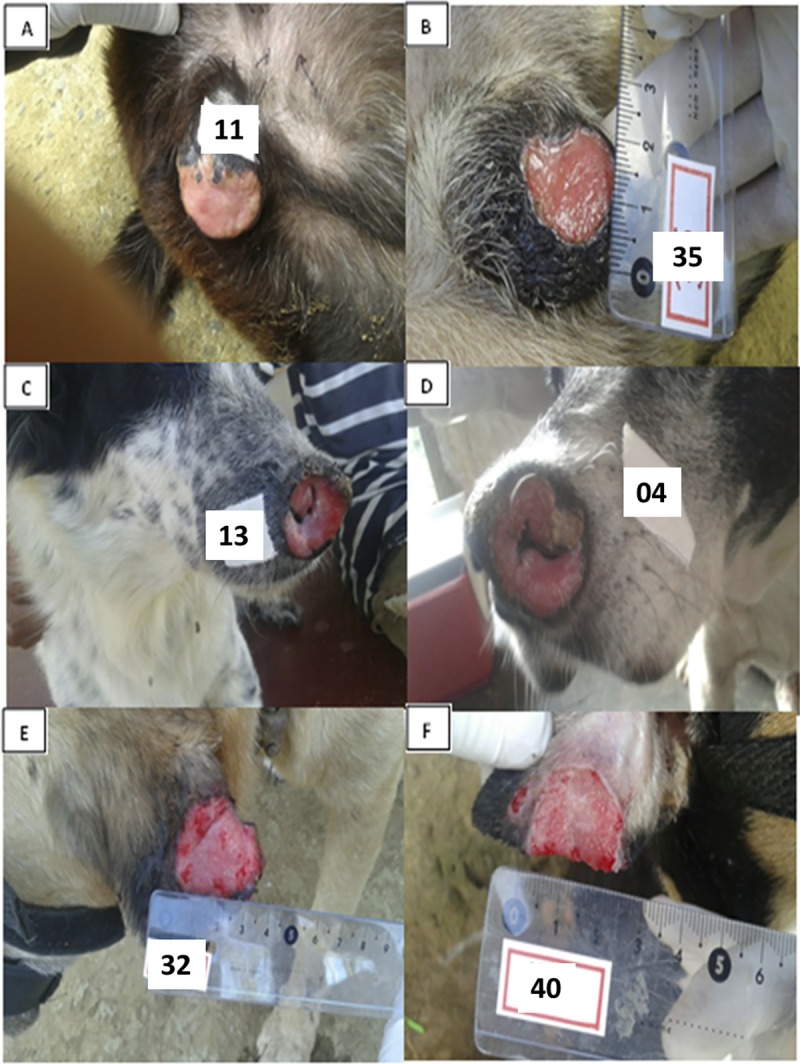
Cutaneous and mucosal lesions caused by *Leishmania (Viannia) braziliensis*. The ulcerated lesions suggestive of ATL were located in the scrotal sac (A and B), muzzle (C and D) and ears (E and F), being, for the most part, single lesions, ulcerated or ulcero-crusted and of chronic evolution.

All the lesions were well limited ulcers with raised borders (Fig 1). The pictures are from six different animals with ulcers located in the scrotal sac (Fig 1A and 1B), in the nose (Fig 1C and 1D) and in the ears (Fig 1E and 1F). In all of these lesions, DNA of *L*. *braziliensis* was documented in the biopsies. The age of the animals presented in these pictures ranged from three years (Fig 1B) to 10 years (Fig 1C).

The sensitivity of all the three tests together to detect exposure to *L*. *braziliensis* was 89%, and the concordance between the tests was high. For this analysis, we used the PCR test as the gold standard. The concordance for PCR and LST and PCR and serology was considered substantial (κ = 0.597 and κ = 0.674, respectively). In the 41 animals with positive PCR, antibodies were detected in 37 (90%) and the LST was positive in 32 (78%). In the 41 animals with positive serology, the LST was positive in 32 (78%). The age of the 54 dogs with at least one positive test was 6 ± 3.2 years and among the seven animals without evidence of exposure was 3 ± 1.9 years (p = 0.02). The illness duration in the two groups was of 210 (90–547) and 75 (38–296) years respectively (p = 0.12).

The histopathology analysis of the cutaneous ulcer border was performed in 35 animals, (Table 2).

**Table 2:** Histopathological findings in dogs with ulcerated lesions.

Histopathological Findings			
	Samples with detected amastigotes	Samples without detected amastigotes	
	N = 26	N = 9	P value
**Inflammation**			
Intense	6 (23%)	1 (11%)	ns
Moderate or mild	20 (73%)	8 (88%)	
**Necrosis**			
Presence	11 (42%)	5 (56%)	ns
Absenc	15 (57%)	4 (44%)	
**Granulomas**			
Presence	2 (8%)	1 (11%)	ns
Absence	24 (92%)	8 (89%)	
**Fibrosis**			
Intense	20 (76%)	7 (78%)	ns
Moderate or mild	6 (24%)	2 (22%)	

Grades of inflammation and fibrosis a ≤ 30 (mild) b ≥ 30 ≤50 (moderate) c ˃ 50 ≤ 100 of fragment

**Fig 2:**
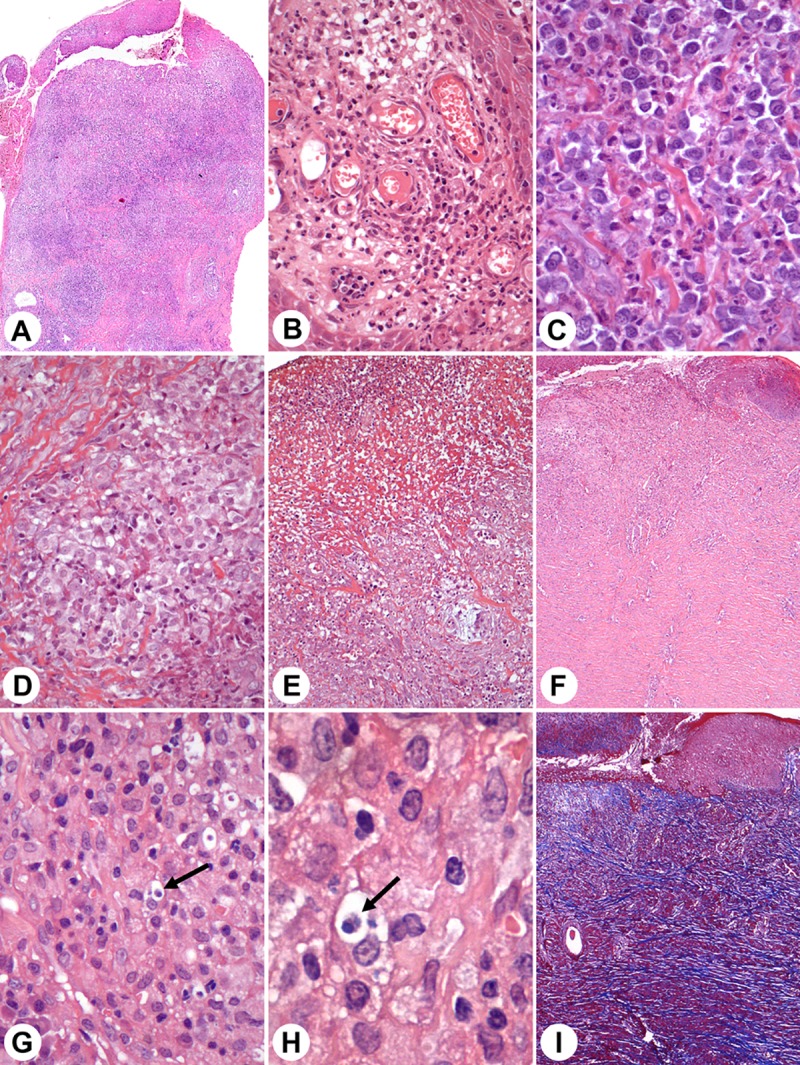
Histopathological findings in canine leishmaniasis ulcers. **(A)** Dog 1 Ulcer with intense inflammation 4X. **(B)** Dog 1 Tissue granulation 20X**. (C)** Dog 5 Intense inflammation, plasma cells 40X. **(D)** Dog 26 Granuloma 20X. **(E)** Dog 1 Necrosis 20X. **(F)** Dog 2 Fibrosis (4X). **(G)** Dog 1 Amastigotes inside macrophage (arrow) 10X. **(H)** Dog 1 Amastigotes inside macrophage (arrow) 100X. **(I)** Dog 2 Fibrosis in blue (same of F) stained by Masson`s Trichrome 4X.

In 26 animals (74%), amastigotes were documented, and in nine (26%) parasites were not detected. However, the histopathologic features were similar in those with or without evidence of amastigotes. In all the animals, there was a chronic inflammation with lymphocytes, plasma cells, macrophages infiltration and granulation tissue. The inflammation was not associated either with the presence of parasites, necrosis and fibrosis were documented (Fig 2).

An inflammation predominantly moderate or mild was observed in 77% of the biopsies with the presence of amastigotes, and a similar finding was detected in 89% of the biopsies negative for parasites. Granuloma was observed in only three (8%) of the animals, two of which were PCR positive. Focal necrosis was observed in 42% and 55% in the biopsies with or without amastigotes, respectively. Presence of fibrosis was observed in 75% of the biopsies, and intense or moderate fibrosis was similar in animals with or without parasites.

In order to check whether *L*. *braziliensis* strains circulating among dogs in Corte de Pedra might be shared by human CL patients in that region, the 600 base pairs long locus that starts at the nucleotide position 425,451 on the parasite’s chromosome 28 (i.e. CHR28/425451) was PCR amplified, cloned, sequenced and compared across a panel of isolates. After alignment of CHR28/425451 sequences, a total of 20 different alleles could be identified in parasites isolated from dogs as determined by their *SNP* and *indel* contents, while seven could be identified in *L*. *braziliensis* obtained from human beings. Overall, 24 different alleles could be enlisted, since parasites from human beings and dogs shared three distinct alleles, which were based on the contents of positions 30, 286 and 545 within the locus that could be described as TT-, CCT and CC*-*. The frequency distribution of seven alleles in *L*. *braziliensis* locus CHR28/425451 among patients and dogs are shown on (Table 3).

**Table 3:** Frequency distribution of seven alleles in *L*. *braziliensis* locus CHR28/425451 among ATL patients and domestic dogs from Corte de Pedra, Bahia, Northeast Brazil.

Allele	Frequency in ATL patients (%)	Frequency in dogs (%)
**TT-**	**35**	**28**
**TTT**	**11**	**0**
**TCT**	**1**	**0**
**TC-**	**2**	**0**
**CT-**	**1**	**0**
**CCT**	**75**	**66**
**CC-**	**11**	**21**

Alleles were defined based on haplotypes of polymorphic nucleotide found in positions 30, 286 and 545 within CHR28/425451.

The frequency of isolates presenting the alleles CCT was high in both dogs and humans, and alleles TT- were present similarly in isolates of both humans and dogs.

## Discussion

The knowledge of wild and domestic reservoirs of *Leishmania* are important to understanding the dynamic of the transmission of this protozoa and to the establishment of control measures, aimed to decrease transmission and consequently decrease the appearance of disease. While the role of dogs in the epidemiology of *L*. *infantum* infection is well known, little is known about the reservoirs of *L*. *braziliensis*. Here, we show that in a highly endemic area of *L*. *braziliensis*, dogs present ulcerated lesions typical of CL, and have evidences of *L*. *brazilensis* infection by documentation of DNA of *L*. *braziliensis*, documentation of parasites in histopathology examination, positive serologic test and or a positive leishmania skin test.

There are previous publications calling attention to the presence of dogs presenting ulcerated lesions with evidence of *Leishmania* infection or even documentation of parasites in bone marrow and spleen of asymptomatics dogs in areas of *L*. *braziliensis* transmission [6, 29, 30]. However, the majority of these studies lack documentation that *L*. *braziliensis* was the causal agent of the infection [8, 31–39]. Here, evaluating 61 dogs with ulcerated lesions, evidence of leishmaniasis infection was observed in 89%, and 67% of the animals had detection of DNA of *L*. *braziliensis*. The clinical characteristic of the lesion was a well limited ulcer with raised borders similar to the ulcers typically found in human CL due to *L*. *braziliensis*. The presence of ulcers mainly in the scrotal sac was likely due to this area being exposed to sandflies bites. The long duration of the disease in dogs differs from what has been observed in humans with *L*. *braziliensis*. The short illness duration in humans could be explained in part by the seek for medical attention and use of therapy for leishmaniasis. But, based in studies in which no therapy or use of placebos were administrated in clinical trials with human cutaneous ulcers caused by *L*. *braziliensis*, the lesions were healed within one year of illness duration [40–43]. In contrast, in this study, the majority of the dogs had illness duration for more than six months and some of them had the disease for more than three to five years. These data support the argument that dogs keep the illness for a long period of time, and the active disease may increase their ability to transmit the infection.

The histopathology features of the dogs with ulcerated lesions was characterized by lymphocytes, plasma cells and macrophages infiltration as it is observed in human CL [6,26]. Nevertheless, they differ from the lesions in humans because they have less inflammation and more vascularization than human lesions. The similarities between the histopathologic findings in animals with positive or negative PCR and in dogs with or without evidence of amastigotes support the hypothesis that likely all the animals in the present study may have been infected with *Leishmania*. The observation that the intensity of the inflammatory reactions was moderate or light in the majority of the animals may be explained by the long illness duration of the disease and the persistence of the lesions for many years.

We have previously shown that *L*. *braziliensis* in Corte de Pedra consists in a complex population made of several different strains of the parasite [18]. We further extended this understanding after cloning and comparing the sequences of several loci in different chromosomes of the *L*. *braziliensis* of Corte de Pedrda [27]. In that study, we detected six loci that presented two or more alleles in that protozoa population. In that and follow-up research, we successfully employed the 500 base-pairs locus that starts at nucleotide position 425,451 of *L*. *braziliensis* chromosome 28 to test association between parasite genotype and outcomes of leishmaniasis [27,44,45]. In the current study, we explored that same locus to determine if parasites that infect the dogs presented genotypes similar to those that infect humans.

The observation that the frequency of the alleles TT- and CCT was similar in *L*. *braziliensis* isolated from dogs and humans suggests that *L*. *braziliensis* from humans and dogs share, at least in part, similar genotype profile in Corte de Pedra. We are aware that in addition to these observations others studies need to be performed to better characterize the importance of dogs and canine TL in the transmission of *L*. *braziliensis*. However, it is relevant that additionally to these findings regarding the similarities between the genotypic characteristics of humans and dogs isolates, active disease or past history of CL was documented in all houses with dogs with TL. Specifically, there were active or past history of CL in 93 individuals living in the 50 houses where canine TL was observed.

This study shows that in an area of *L*. *brazilensis* transmission dogs presenting cutaneous ulcers are likely infected by *L*. *brazilensis*. The clinical and histologic features of canine CL were similar to the observed features in humans, but dogs may remain with the disease for a longer period of time. The occurrence of humans with CL or previous history of CL, and dogs with canine CL in the same house and the similarities between the parasites isolates from dogs and humans strongly argue in favor of the possibility that dogs participate in the transmission of *L*. *braziliensis*.

## Supporting information

S1 Fig Sequences of alleles found in the 500 base-pairs locus on *Leishmania braziliensis* chromosome 28 (CHR28/425451). Two major groups of alleles were found: A and B. At the top of each group the complete sequence of one reference allele is displayed (allele 1 for group A; allele 10 for group B). In the remainder alleles dots (.) represent nucleotide positions that present the same content of the reference alleles, letters consist in nucleotides that differ from those found at the same position in the reference alleles, minuses (-) represent indels between alleles at the specified reference allele position. Nucleotide bases: A is adenine, C is cytosine, T is thymine, G is guanine.
(JPG)
