# Supplementary figures and images for: Clinical and histopathologic features of canine tegumentary leishmaniasis and the molecular characterization of Leishmania braziliensis in dogs
Source: PLoS Negl Trop Dis. 2019 Jul 16;13(7):e0007532. doi: 10.1371/journal.pntd.0007532 (PMC6634374; doi:10.1371/journal.pntd.0007532)

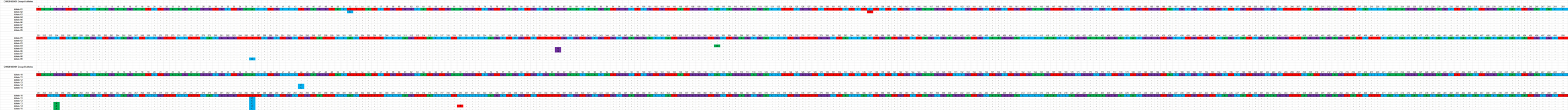

Supplement: S1 Fig — Two major groups of alleles were found: A and B. At the top of each group the complete sequence of one reference allele is displayed (allele 1 for group A; allele 10 for group B). In the remainder alleles dots (.) represent nucleotide positions that present the same content of the reference alleles, letters consist in nucleotides that differ from those found at the same position in the reference alleles, minuses (-) represent indels between alleles at the specified reference allele position. Nucleotide bases: A is adenine, C is cytosine, T is thymine, G is guanine. (JPG) [file pntd.0007532.s001.jpg]
